# Impact of heart rate on eGFR decline in ischemic stroke patients

**DOI:** 10.1093/ckj/sfae387

**Published:** 2024-11-30

**Authors:** Jiann-Der Lee, Ya-Wen Kuo, Chuan-Pin Lee, Yen-Chu Huang, Meng Lee, Tsong-Hai Lee

**Affiliations:** Department of Neurology, Chiayi Chang Gung Memorial Hospital, Chiayi, Taiwan; College of Medicine, Chang Gung University, Taoyuan, Taiwan; Department of Neurology, Chiayi Chang Gung Memorial Hospital, Chiayi, Taiwan; Department of Nursing, Chang Gung University of Science and Technology, Chiayi Campus, Chiayi, Taiwan; Health Information and Epidemiology Laboratory, Chiayi Chang Gung Memorial Hospital, Chiayi, Taiwan; Department of Neurology, Chiayi Chang Gung Memorial Hospital, Chiayi, Taiwan; College of Medicine, Chang Gung University, Taoyuan, Taiwan; Department of Neurology, Chiayi Chang Gung Memorial Hospital, Chiayi, Taiwan; College of Medicine, Chang Gung University, Taoyuan, Taiwan; College of Medicine, Chang Gung University, Taoyuan, Taiwan; Department of Neurology, Linkou Chang Gung Memorial Hospital, Taoyuan, Taiwan

**Keywords:** glomerular filtration rate, heart rate, ischemic stroke

## Abstract

**Background:**

Resting heart rate is a potent predictor of various renal outcomes. However, the decline rate of renal function in ischemic stroke patients is not well defined. We tested the association of heart rate with estimated eGFR decline and the composite renal outcomes in patients with recent ischemic stroke.

**Methods:**

The data of 9366 patients with ischemic stroke with an eGFR of ≥30 mL/min/1.73 m^2^ were retrieved from the Chang Gung Research Database. Mean initial in-hospital heart rates were averaged and categorized into 10-beats-per-minute (bpm) increments. The outcomes were the annualized change in eGFR across the heart rate subgroups and composite renal outcomes, namely a ≥40% sustained decline in eGFR, end-stage renal disease, or renal death. Generalized estimating equation models were used to determine the annualized change in eGFR and Cox proportional hazards regression models were used to estimate the relative hazard of composite renal outcomes by referencing the subgroup with a heart rate of <60 bpm.

**Results:**

The annual eGFR decline in the patients with a mean heart rate of <60, 60–69, 70–79, 80–89, and ≥90 bpm was 2.12, 2.49, 2.83, 3.35, and 3.90 mL/min/1.73 m^2^, respectively. Compared with the reference group, the adjusted hazard ratios for composite renal outcomes were 1.17 [95% confidence interval (CI), 0.89–1.53), 1.54 (95% CI, 1.19–2.00), 1.72 (95% CI, 1.30–2.28), and 1.84 (95% CI, 1.29–2.54] for the patients with a heart rate of 60–69, 70–79, 80–89, and ≥90 bpm, respectively. In the subgroup analysis, the associations between higher heart rate and both eGFR decline and composite renal outcomes were more evident and statistically significant in patients without atrial fibrillation.

**Conclusions:**

A higher heart rate is associated with a faster rate of eGFR decline and an increased risk of composite renal outcomes after ischemic stroke, particularly in patients without atrial fibrillation. These results underscore the importance of heart rate monitoring and management in ischemic stroke patients in sinus rhythm to potentially mitigate renal function decline. Further studies are needed to explore this relationship in patients with atrial fibrillation and across different ethnic groups.

## Graphical Abstract

**Graphical Abstract:**
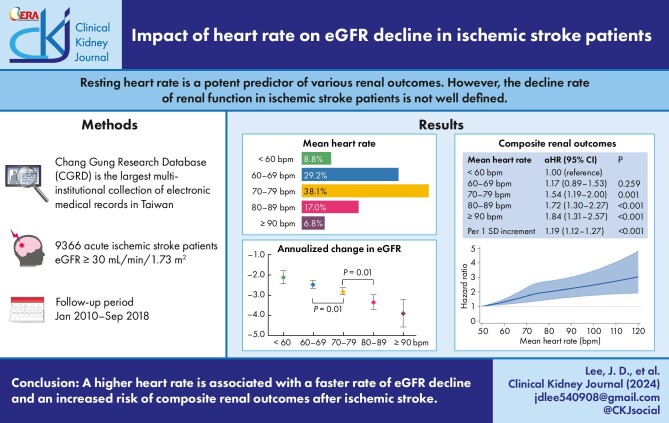

KEY LEARNING POINTS **What was known:**

- Ischemic stroke is a leading cause of disability and mortality globally and significantly increases the risk of CKD and renal function decline.
- Patients with CKD face a heightened risk of stroke, and even slight decreases in kidney function, measured by eGFR, elevate the risk of cardiovascular and cerebrovascular diseases.
- While various risk factors for renal disease have been identified, the role of heart rate in renal function decline has been largely overlooked, despite its associations with hemodynamics, autonomic regulation, and inflammation.

**This study adds:**

- A higher resting heart rate is significantly associated with an increased risk of composite renal outcomes, including a higher rate of eGFR decline and adverse renal events.
- This study provides evidence that heart rate is an important prognostic factor for renal outcomes in ischemic stroke patients, emphasizing the need for heart rate management in this population.

**Potential impact:**

- Resting heart rate measurement can be integrated into routine risk assessments for patients with ischemic stroke to identify those at higher risk of rapid eGFR decline and adverse renal outcomes.
- Monitoring and managing heart rate in ischemic stroke patients may help to slow kidney function decline and improve long-term renal and cardiovascular outcomes.

## INTRODUCTION

Ischemic stroke is a prevalent and severe neurovascular condition, leading to significant disability and death globally. Stroke is also a significant and independent risk factor for CKD, renal function decline, and end-stage renal disease [1]. Conversely, CKD patients face a higher risk of all stroke subtypes [2]. Additionally, renal dysfunction significantly increases the risk of post-stroke complications and death [3]. Even a slight decrease in kidney function, which is assessed on the basis of the eGFR, is associated with a significantly increased risk of comorbid conditions such as cardiovascular and cerebrovascular disease [4, 5]. The kidneys and brain are particularly susceptible to vascular damage because the vascular regulation of their microvasculature is anatomically and functionally similar [6]. Along with preventing further cardiovascular events, renal protection is also crucial in the care of patients with ischemic stroke.

The complex associations of heart rate with hemodynamics [7], autonomic regulation [8], and inflammation [9] are likely to contribute to the association between heart rate and renal function decline. Emerging evidence from large cohort studies has revealed that a higher heart rate is significantly associated with an accelerated progression of CKD and an increased risk of end-stage renal disease [10, 11]. In ischemic stroke, the acute sympathetic surge and resultant increase in heart rate may exacerbate renal hypoperfusion and oxidative stress, accelerating eGFR decline [12–14]. Additionally, heightened heart rate could influence vascular stiffness and endothelial dysfunction, which are known to negatively affect renal function [15]. Therefore, understanding the specific relationship between heart rate and eGFR trajectory in this patient population is crucial for developing targeted interventions to protect kidney function.

To our knowledge, no research has investigated resting heart rate as a determinant of the average rate of eGFR decline in ischemic stroke patients. In the present study, we tested the hypothesis that the initial in-hospital heart rate of patients with recent ischemic stroke is associated with their rate of eGFR decline and composite renal outcomes.

## MATERIALS AND METHODS

### Study design and population

For the present retrospective cohort study, we retrieved patient data from the Chang Gung Research Database, which is the largest multi-institutional collection of electronic medical records in Taiwan. The Chang Gung Research Database comprises data from all records of emergency services, inpatient and outpatient visits, including electronic medical records, demographic characteristics, imaging reports, laboratory results, pharmacy dispensing details, nursing records, and discharge summaries. The overall coverage rate of the Chang Gung Research Database for patients in Taiwan was 21% for outpatients and 12% for inpatients [16].

### Inclusion and exclusion criteria

**Figure 1:**
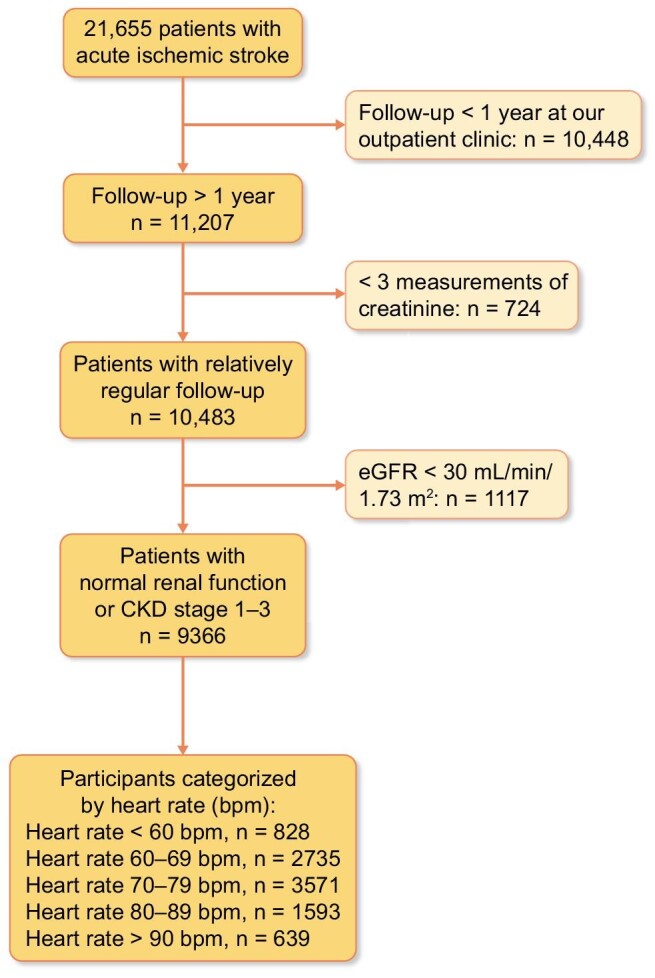
Flow chart of patient selection.

Patients with acute ischemic stroke accrued consecutively between 1 January 2010 and 30 September 2018 were included. The process for selecting patients with recent ischemic stroke was described in our previous study [17]. The exclusion criteria were patients (i) with follow-up of <1 year at our outpatient clinic; (ii) with fewer than three measurements of creatinine; and (iii) with a baseline eGFR of <30 mL/min/1.73 m². The study design and patient selection process are illustrated in Fig. 1.

Data pertaining to primary demographic and clinical characteristics were retrieved, including stroke severity as assessed using the claims-based stroke severity index (SSI). The stroke severity data (i.e. SSI scores) were subsequently converted to National Institutes of Health Stroke Scale (NIHSS) scores by applying the following equation: estimated NIHSS score = 1.1722 × SSI − 0.7533 [18]. The eGFR of the enrolled patients was determined using the Chronic Kidney Disease Epidemiology Collaboration equation because it has been suggested to be more accurate than the Modification of Diet in Renal Disease Study equation, particularly for populations without CKD [19].

Data pertaining to the enrolled patients’ height, body weight, systolic blood pressure, diastolic blood pressure, heart rate, lipid profile, and levels of creatinine, alanine aminotransferase, and glycated hemoglobin after admission were retrieved from the database. The vital sign values recorded in this study were measured during the patients' hospitalization by well-trained nursing staff in our healthcare system, following procedures based on the recommendations of the American Heart Association [20]. To minimize bias, the mean heart rate, systolic blood pressure, and diastolic blood pressure in the first 3 days of hospitalization were used instead of a single measurement value for further analysis.

### Study outcomes

The primary renal outcome was the annualized change in kidney function, which was measured by obtaining a slope of eGFR values. The prespecified secondary renal outcome was a composite comprising a sustained eGFR decline of ≥40%, end-stage renal disease, or renal death. A sustained eGFR decline was defined as a decline lasting at least 28 days, and end-stage renal disease was defined as a sustained eGFR of <15 mL/min/1.73 m^2^ or a condition requiring chronic dialysis or renal transplantation. The clinical data extracted from the Chang Gung Research Database are linked to the National Registry of Deaths operated by the Ministry of Health and Welfare in Taiwan pertained to the period 1 January 2010 to 31 December 2018. The main causes of death were classified using the International Classification of Diseases, 10th Revision, Clinical Modification (ICD-10-CM), and renal death was classified using the ICD-10-CM codes N00–N07 and N17–N19 [21].

### Statistics

Quantitative variables are expressed as means (standard deviations) and medians (interquartile range), and categorical variables are expressed as numbers (percentages). The data on primary demographic and clinical characteristics were stratified by heart rate [i.e. <60 , 60–70, 70–79, 80–89, and ≥90 beats per minute (bpm)]. To identify the differences between the heart rate subgroups, the Kruskal–Wallis rank test and *χ*^2^ test were performed for continuous and categorical data, respectively. We employed population average models based on generalized estimating equations to determine the annualized change in eGFR across heart rate subgroups; the equation for calculating the annualized change in eGFR after adjustment for potential confounding variables is as follows [22]:


\begin{eqnarray*}
{\mathrm{eGFR}}\sim\beta i &+& \gamma i \times {\mathrm{years}} + {\mathrm{effects}}\,{\mathrm{of}}\,{\mathrm{confounding}}\,{\mathrm{variables}}\nonumber\\
&+& {\mathrm{error}}\,{\mathrm{term}}
\end{eqnarray*}

where *βi* is the average baseline eGFR of each heart rate subgroup, *γi* is the average eGFR slope (annualized change) of each heart rate subgroup, and *i* = 1, 2, 3, 4, and 5 represent the subgroups comprising patients with a heart rate of <60, 60–69, 70–79, 80–89, and ≥90 bpm, respectively.

We performed Cox proportional hazards regression to estimate the relative hazard of composite renal outcomes. Analyses were performed using heart rate as both a continuous and categorical variable. In addition to crude hazard ratios (HRs), adjusted HRs and 95% confidence intervals (CIs) were calculated by referencing the subgroup with a heart rate of <60 bpm and estimated after adjustment for potential confounding factors.

We selected the covariates before analysis based on their established plausibility to confound the association between heart rate and eGFR decline or composite renal outcomes, as identified in prior research [23]. We constructed three multivariable models to assess multiple groups of potential confounding variables. Model 1 was adjusted for age and sex. Model 2 was adjusted for the variables in model 1 and stroke severity, BMI, hypertension, diabetes, dyslipidemia, atrial fibrillation, coronary artery disease, congestive heart failure, history of cancer, smoking status, total cholesterol level, triglyceride level, alanine aminotransferase level, glycated hemoglobin level, mean systolic blood pressure, and mean diastolic blood pressure. Model 3 was adjusted for the variables in model 2 plus the use of angiotensin converting enzyme inhibitors (ACEIs)/angiotensin II receptor blockers (ARBs), antithrombotics, β-blockers, parasympathomimetics, and statins. Unless specified, all presented results were generated using the fully adjusted model (model 3). Data analysis was conducted without the imputation of missing data. Variables with missing data were classified under a missing data category to minimize the effect of missing data in the analysis.

A Cox model and restricted cubic spline smoothing technique were employed to explore the overall trend of risks for composite renal outcomes throughout the heart rate ranges. Subgroup analyses of composite renal outcomes were performed by using heart rate as a continuous variable. The hazard ratios and 95% confidence intervals for each subgroup were calculated for every increment of 1 standard deviation in heart rate. All analyses were performed using SAS (version 9.4, Cary, NC, USA) and R software version 4.2.0 (R Foundation for Statistical Computing, Vienna, Austria).

The study was conducted in accordance with the Declaration of Helsinki and local ethics approval was obtained.

## RESULTS

### Baseline characteristics

In total, 9366 adult patients with recent ischemic stroke were included in the analysis (mean age, 66.6 ± 12.3 years; men, 63.8%). The patients’ mean eGFR was 81.5 ± 21.0 mL/min/1.73 m^2^. Their mean systolic blood pressure and diastolic blood pressure were 151.3 ± 19.0 and 85.7 ± 10.7 mmHg, respectively, and their mean heart rate was 74.84 ± 11.27 bpm. The median number of eGFR readings used to analyze the rate of eGFR decline was 6 (interquartile range, 4–7). The demographic data and baseline characteristics of the overall patient cohort and the heart rate subgroups (categorized in 10-bpm increments) are presented in Table 1.

**Table 1:** Demographic and baseline characteristics of the overall cohort stratified by mean heart rate.

	**Mean heart rate (bpm)**						
	**Total (*n* = 9366)**	**<60** **(*n* = 828)**	**60–69 (*n* = 2735)**	**70–79 (*n* = 3571)**	**80–89 (*n* = 1593)**	**≥90 (*n* = 639)**	***P* value**
Age (years)							0.007
Mean (SD)	66.60 (12.25)	67.73 (10.74)	66.57 (11.68)	66.26 (12.32)	66.54 (13.16)	67.30 (13.59)	
Median (Q1, Q3)	67.00 (59.00, 76.00)	69.00 (61.00, 75.00)	67.00 (59.00, 75.00)	67.00 (58.00, 76.00)	67.00 (59.00, 77.00)	69.00 (58.00, 78.00)	
Male (%)	5979 (63.84%)	601 (72.58%)	1821 (66.58%)	2252 (63.06%)	933 (58.57%)	372 (58.22%)	<0.001
Stroke severity							<0.001
Mild (eNIHSS < 6)	6706 (71.60%)	658 (79.47%)	2176 (79.56%)	2641 (73.96%)	997 (62.59%)	234 (36.62%)	
Moderate (eNIHSS 6–13)	1799 (19.21%)	130 (15.70%)	434 (15.87%)	695 (19.46%)	381 (23.92%)	159 (24.88%)	
Severe (eNIHSS > 13)	861 (9.19%)	40 (4.83%)	125 (4.57%)	235 (6.58%)	215 (13.50%)	246 (38.50%)	
BMI (kg/m^2^)							0.02
Mean (SD)	25.21 (4.22)	24.80 (3.83)	25.27 (4.10)	25.35 (4.20)	25.17 (4.36)	25.00 (4.79)	
Median (Q1, Q3)	24.80 (22.59, 27.43)	24.61 (22.48, 27.16)	24.93 (22.70, 27.36)	25.00 (22.77, 27.58)	24.61 (22.31, 27.47)	24.34 (22.21, 27.34)	
Hypertension	7166 (76.51%)	637 (76.93%)	2117 (77.40%)	2713 (75.97%)	1233 (77.40%)	466 (72.93%)	0.1
Diabetes mellitus	3867 (41.29%)	238 (28.74%)	963 (35.21%)	1553 (43.49%)	794 (49.84%)	319 (49.92%)	<0.001
Dyslipidemia	4524 (48.30%)	416 (50.24%)	1368 (50.02%)	1751 (49.03%)	732 (45.95%)	257 (40.22%)	<0.001
Atrial fibrillation	1231 (13.14%)	72 (8.70%)	252 (9.21%)	399 (11.17%)	312 (19.59%)	196 (30.67%)	<0.001
Coronary artery disease	794 (8.48%)	68 (8.21%)	227 (8.30%)	286 (8.01%)	148 (9.29%)	65 (10.17%)	0.3
Congestive heart failure	373 (3.98%)	24 (2.90%)	75 (2.74%)	140 (3.92%)	79 (4.96%)	55 (8.61%)	<0.001
History of cancer	522 (5.57%)	52 (6.28%)	145 (5.30%)	193 (5.40%)	81 (5.08%)	51 (7.98%)	0.06
Current smoker	2764 (29.51%)	307 (37.08%)	906 (33.13%)	978 (27.39%)	399 (25.05%)	174 (27.23%)	<0.001
Total cholesterol (mg/dL)							0.04
Mean (SD)	180.55 (42.47)	177.80 (41.34)	180.46 (39.51)	181.52 (41.63)	180.46 (45.98)	179.34 (50.86)	
Median (Q1, Q3)	177.27 (152.24, 204.32)	174.27 (151.23, 200.31)	177.27 (154.24, 203.31)	178.28 (153.24, 205.32)	177.27 (150.23, 203.31)	173.77 (147.48, 206.32)	
Triglyceride (mg/dL)							<0.001
Mean (SD)	137.20 (101.31)	126.89 (85.99)	135.48 (84.03)	139.09 (104.48)	143.37 (119.77)	132.05 (116.82)	
Median (Q1, Q3)	114.10 (82.07, 162.14)	109.10 (80.07, 152.13)	115.10 (84.07, 161.14)	116.10 (83.07, 165.15)	112.10 (82.07, 167.15)	103.09 (75.32, 150.88)	
ALT (U/L)							0.7
Mean (SD)	27.62 (36.52)	26.15 (24.39)	27.15 (24.25)	27.08 (20.29)	29.69 (71.63)	29.36 (33.81)	
Median (Q1, Q3)	21.50 (16.00, 30.00)	21.00 (16.00, 29.00)	21.00 (16.00, 30.00)	22.00 (16.00, 31.00)	22.00 (16.00, 31.00)	22.00 (16.00, 32.00)	
HbA1c (%)							<0.001
Mean (SD)	6.93 (1.90)	6.41 (1.34)	6.69 (1.69)	6.97 (1.86)	7.28 (2.18)	7.47 (2.40)	
Median (Q1, Q3)	6.20 (5.70, 7.50)	5.90 (5.70, 6.50)	6.00 (5.70, 7.10)	6.20 (5.70, 7.60)	6.40 (5.80, 8.15)	6.40 (5.80, 8.40)	
Mean SBP (mmHg)							<0.001
Mean (SD)	151.31 (18.99)	153.48 (20.51)	152.31 (19.16)	149.96 (18.40)	152.03 (19.05)	149.95 (18.77)	
Median (Q1, Q3)	150.11 (138.00, 164.00)	152.66 (139.44, 168.09)	151.38 (138.64, 165.20)	148.20 (137.18, 162.05)	151.20 (139.17, 165.15)	149.05 (136.91, 163.16)	
Mean DBP (mmHg)							<0.001
Mean (SD)	85.69 (10.73)	83.09 (10.76)	85.07 (10.73)	85.61 (10.21)	87.68 (11.24)	87.13 (11.21)	
Median (Q1, Q3)	85.00 (78.36, 92.56)	82.94 (75.64, 89.71)	84.57 (77.50, 92.00)	84.64 (78.62, 91.87)	87.38 (80.01, 95.33)	86.95 (79.68, 94.25)	
Medications at discharge							
ACEI/ARB	5483 (58.54%)	473 (57.13%)	1589 (58.10%)	2076 (58.13%)	960 (60.26%)	385 (60.25%)	0.4
Antithrombotic	9095 (97.11%)	811 (97.95%)	2667 (97.51%)	3472 (97.23%)	1541 (96.74%)	604 (94.52%)	<0.001
β-Blocker	2909 (31.06%)	202 (24.40%)	800 (29.25%)	1081 (30.27%)	555 (34.84%)	271 (42.41%)	<0.001
Parasympathomimetic	323 (3.45%)	23 (2.78%)	70 (2.56%)	110 (3.08%)	78 (4.90%)	42 (6.57%)	<0.001
Statin	4683 (50.00%)	435 (52.54%)	1382 (50.53%)	1789 (50.10%)	766 (48.09%)	311 (48.67%)	0.3
Baseline eGFR (mL/min per 1.73 m^2^)							<0.001
Mean (SD)	81.47 (21.02)	78.98 (19.96)	81.20 (20.35)	81.31 (20.66)	82.47 (21.97)	84.19 (24.19)	
Median (Q1, Q3)	84.22 (66.32, 96.79)	82.16 (64.57, 94.61)	83.87 (66.68, 96.60)	84.05 (66.24, 96.58)	85.38 (67.13, 97.54)	88.25 (66.02, 100.53)	
Baseline eGFR categories							<0.001
≥100	1814 (19.37%)	116 (14.01%)	512 (18.72%)	675 (18.90%)	344 (21.59%)	167 (26.13%)	
≥90, <100	1848 (19.73%)	165 (19.93%)	542 (19.82%)	708 (19.83%)	302 (18.96%)	131 (20.50%)	
≥60, <90	4027 (43.00%)	384 (46.38%)	1204 (44.02%)	1546 (43.29%)	672 (42.18%)	221 (34.59%)	
≥30, <60	1677 (17.91%)	163 (19.69%)	477 (17.44%)	642 (17.98%)	275 (17.26%)	120 (18.78%)	

Q, quartile; eNIHSS, estimated National Institute of Health Stroke Scale; ALT, alanine aminotransferase; HbA1c, glycated hemoglobin; SBP, systolic blood pressure; DBP, diastolic blood pressure.

### Primary outcome: association of initial in-hospital heart rate with eGFR decline

**Figure 2:**
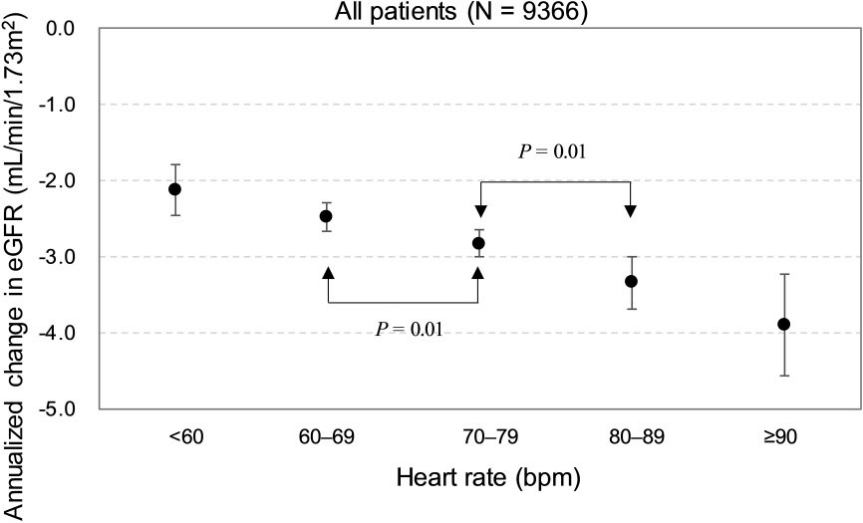
Annualized change of average eGFR in different heart rate subgroup.

Supplementary Fig. S1 presents the longitudinal evolution of eGFR in each heart rate subgroup. The average annual rate of eGFR decline was lower among the patients with a lower heart rate. After adjustments were made for potential confounding factors, the average annual decrease in the patients’ eGFR was as follows: patients with a mean heart rate of <60 bpm, 2.12 mL/min/1.73 m^2^; patients with a mean heart rate of 60–69 bpm, 2.49 mL/min/1.73 m^2^; patients with a mean heart rate of 70–79 bpm, 2.83 mL/min/1.73 m^2^; patients with a mean heart rate of 80–89 bpm, 3.35 mL/min/1.73 m^2^; and patients with a mean heart rate of ≥90 bpm, 3.90 mL/min/1.73 m^2^ (Fig. 2 and Supplementary Table S1). Through *post hoc* analyses the average annual decline in eGFR was revealed to be significantly different between the heart rate 60–690- and 70–79-bpm subgroups and the 70–79- and 80–89-bpm subgroups (Fig. 2). A higher heart rate was revealed to be associated with a higher rate of eGFR decline.

**Figure 3:**
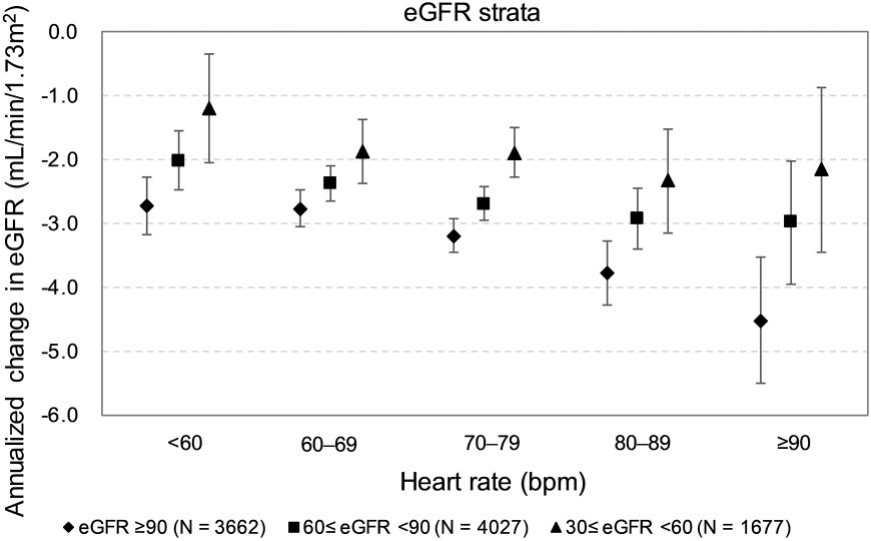
Annualized change of average eGFR in different heart rate subgroups according to baseline eGFR strata.

Figure 3 presents the average annual rate of eGFR decline of each heart rate subgroup based on the baseline eGFR strata (≥90 mL/min/1.73 m^2^, <90 and ≥60 mL/min/1.73 m^2^, and <60 and ≥ 30 mL/min/1.73 m^2^). Overall, the patients with an eGFR of ≥90 mL/min/1.73 m^2^ experienced an accelerated eGFR decline when they had a higher heart rate (Fig. 3 and Supplementary Table S2).

### Secondary outcome: association of initial in-hospital heart rate with composite renal outcomes

During the follow-up period, which had a median duration of 4 years, 1099 patients met the criteria for composite renal outcomes. The calculated annual event rates for composite renal outcomes and the crude and adjusted hazard ratios for the mean heart rate of each subgroup are listed in Table 2. Compared with the reference group (mean heart rate of <60 bpm), the adjusted hazard ratio for composite renal outcomes in model 3 was 1.17 (95% CI, 0.89–1.53) for the patients with a mean heart rate of 60–69 bpm, 1.54 (95% CI, 1.19–2.00) for those with a mean heart rate of 70–79 bpm, 1.72 (95% CI, 1.30–2.28) for those with a mean heart rate of 80–89 bpm, and 1.84 (95% CI, 1.29–2.54) for those with a mean heart rate of ≥90 bpm.

**Table 2:** Association of heart rate with the risk of composite renal outcomes.

		**Incidence rate**	**Hazard ratio (95% CI**)							
**Heart rate (bpm)**	**Number of events (%)**	**(per 100 person-years)**	**Unadjusted**	***P* value**	**Model 1**	***P* value**	**Model 2**	***P* value**	**Model 3**	***P* value**
<60	68 (8.21%)	2.00 (1.58–2.54)	1.00 (reference)		1.00 (reference)		1.00 (reference)		1.00 (reference)	
≥60 and <70	261 (9.54%)	2.35 (2.08–2.65)	1.17 (0.90–1.53)	0.2	1.20 (0.92–1.56)	0.2	1.19 (0.91–1.56)	0.2	1.17 (0.89–1.53)	0.3
≥70 and <80	459 (12.85%)	3.04 (2.78–3.33)	1.51 (1.17–1.94)	0.002	1.55 (1.20–2.00)	0.001	1.57 (1.21–2.04)	0.001	1.54 (1.19–2.00)	0.001
≥80 and <90	225 (14.12%)	3.79 (3.33–4.32)	1.93 (1.47–2.53)	<0.001	1.99 (1.51–2.61)	<0.001	1.76 (1.33–2.33)	<0.001	1.72 (1.30–2.27)	<0.001
≥90	86 (13.46%)	4.02 (3.26–4.97)	2.09 (1.52–2.88)	<0.001	2.14 (1.55–2.94)	<0.001	1.88 (1.34–2.63)	<0.001	1.84 (1.31–2.57)	<0.001
Per 1 SD increment			1.25 (1.18–1.33)	<0.001	1.26 (1.18–1.33)	<0.001	1.20 (1.12–1.28)	<0.001	1.19 (1.12–1.27)	<0.001

Model 1 is adjusted for age and sex.

Model 2 is adjusted for the variables in model 1 plus stroke severity, BMI, hypertension, diabetes, dyslipidemia, atrial fibrillation, coronary artery disease, congestive heart failure, history of cancer, smoking status, total cholesterol, triglycerides, alanine aminotransferase, glycated hemoglobin, systolic blood pressure, and diastolic blood pressure.

Model 3 is adjusted for the variables in model 2 plus ACEi/ARB use, antithrombotic use, β-blocker use, parasympathomimetic use, and statin use.

1 SD = 10.41 bpm.

Age; male sex; estimated NIHSS score; diabetes mellitus; history of coronary artery disease, congestive heart failure, or cancer before admission; current smoking habit; high levels of triglyceride glycated hemoglobin, and systolic blood pressure; and use of ACEIs/ARBs were all revealed to be positively associated with the risk of composite renal outcomes. Conversely, a high BMI and high levels of alanine aminotransferase and diastolic blood pressure exhibited protective effects against composite renal outcomes (Supplementary Table S3).

When the patient results were stratified by baseline eGFR level, those with an eGFR of ≥60 mL/min/1.73 m^2^ exhibited an increased risk of composite renal outcomes when they had a higher heart rate, and those with an eGFR of <60 mL/min/1.73 m^2^ did not exhibit a significantly different risk of composite renal outcomes relative to the other heart rate subgroups (Table 3).

**Table 3:** Association of heart rate with the risk of composite renal outcomes in different heart rate subgroups according to baseline eGFR strata.

	**Adjusted hazard ratio (95% confidence interval)**					
**Variable**	**eGFR ≥ 90**		**60 ≤ eGFR < 90**		**30 ≤ eGFR < 60**	
**Heart rate (bpm)**		***P* value**		***P* value**		***P* value**
<60	1.00 (reference)		1.00 (reference)		1.00 (reference)	
≥60 and <70	1.40 (0.75–2.59)	0.3	1.14 (0.78–1.69)	0.5	1.11 (0.68–1.79)	0.7
≥70 and <80	2.01 (1.10–3.67)	0.02	1.55 (1.06–2.26)	0.02	1.18 (0.74–1.91)	0.5
≥80 and <90	2.48 (1.32–4.64)	0.01	1.68 (1.11–2.53)	0.01	1.38 (0.82–2.33)	0.2
≥90	2.37 (1.17–4.82)	0.02	1.98 (1.21–3.26)	0.01	1.32 (0.67–2.59)	0.4

Hazard ratios are adjusted for age, sex, stroke severity, BMI, hypertension, diabetes, dyslipidemia, atrial fibrillation, coronary artery disease, congestive heart failure, history of cancer, smoking status, total cholesterol, triglyceride, alanine aminotransferase, glycated hemoglobin, systolic blood pressure, diastolic blood pressure, ACEi/ARB use, antithrombotic use, β-blocker use, parasympathomimetic use, and statin use.

**Figure 4:**
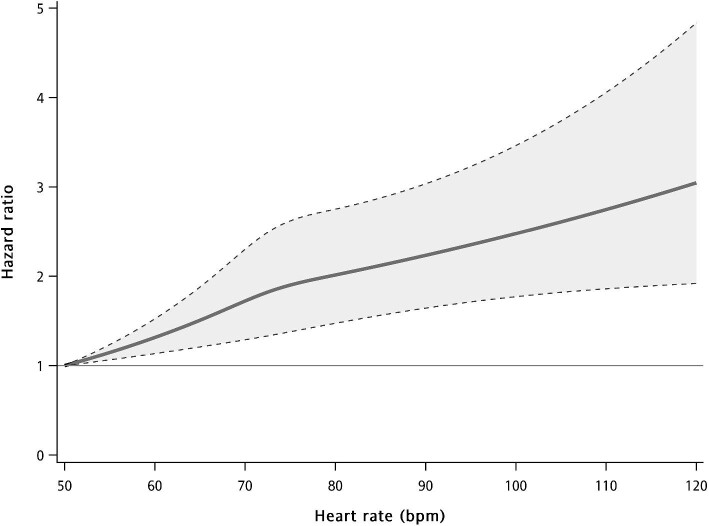
Restricted cubic splines are represented for the associations between initial in-hospital heart rate levels and composite renal outcome (≥40% sustained decline in eGFR or end-stage renal disease or renal death). The analyses were adjusted for all of the variables in the fully adjusted model.

After multiple adjustments were made for potential confounding factors, a higher initial in-hospital heart rate was revealed to be significantly and continuously associated with an increased hazard ratio for composite renal outcomes (Fig. 4).

### Subgroup and sensitivity analyses

**Figure 5:**
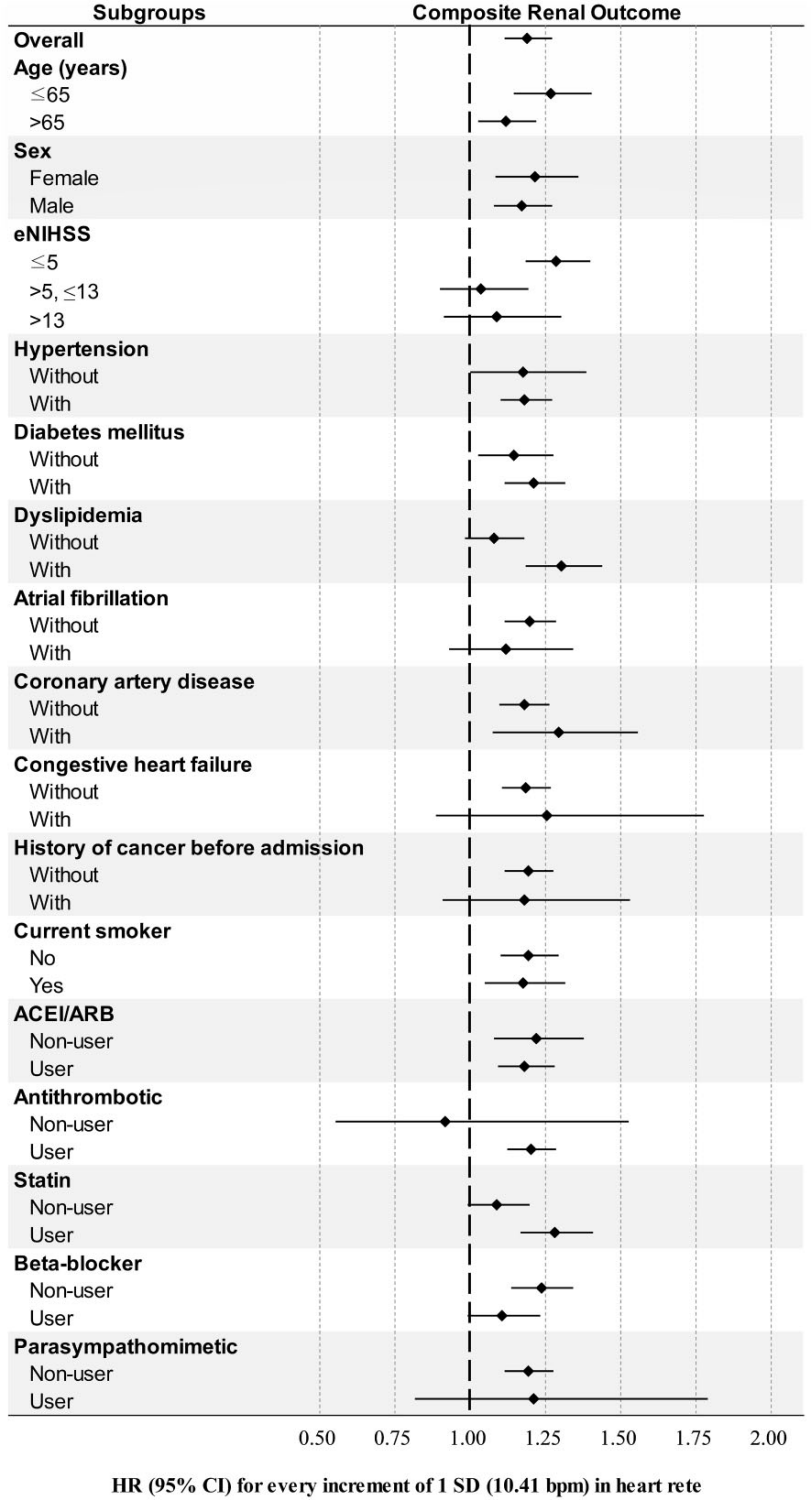
Subgroup analyses of composite renal outcomes for every increment of 1 SD in heart rate (10.41 bpm). eNIHSS, estimated NIHSS.

The subgroup analysis results for composite renal outcomes are presented in Fig. 5. They revealed an association between mean heart rate and the composite renal outcomes in most subgroups, indicating a consistent relationship between initial in-hospital heart rate and composite renal outcomes. However, in patients with atrial fibrillation no significant association was observed between heart rate and renal prognosis, including both eGFR decline (Supplementary Table S4 and Supplementary Figure S2) and composite renal outcomes (Fig. 5). This suggests that the prognostic value of heart rate for renal outcomes may be limited to patients without atrial fibrillation.

## DISCUSSION

In this retrospective cohort study of 9366 patients with recent ischemic stroke, we identified an association between a higher initial in-hospital heart rate and a faster rate of eGFR decline. A higher heart rate was also associated with an increased risk of composite renal outcomes. This result provides novel insights into the effect of heart rate on the decline of renal function and the progression of kidney disease in a population with recent cerebral ischemic events.

Cross-talk occurs between the brain and kidneys after stroke, and stroke may lead to kidney dysfunction, which can adversely influence patient outcomes [24]. CKD raises the risk of recurrent stroke by 50% in those with previous lacunar stroke [25], and there is a graded association between declining eGFR levels and increased risk of recurrent stroke in patients with acute ischemic stroke [26]. Therefore, although most of the patients who experienced a recent ischemic stroke exhibited preserved renal function (82.1% of the patients with an eGFR of ≥60 mL/min/1.73 m^2^ in the present study), those with a high heart rate still required special attention because of their accelerated eGFR decline (Fig. 3), which could have increased the risk of recurrent stroke in this subgroup of patients.

Elevated heart rate can contribute to eGFR decline through several interconnected mechanisms. Hemodynamically, increased heart rate leads to higher cardiac output and blood pressure, causing glomerular hyperfiltration and subsequent glomerular damage [7, 27]. This is compounded by the activation of the sympathetic nervous system, which induces vasoconstriction and raises renal vascular resistance, reducing renal blood flow [8, 28]. Sympathetic overactivity also stimulates the renin–angiotensin–aldosterone system, further harming renal function [29]. Additionally, an elevated heart rate was revealed to be associated with cellular signaling events resulting in vascular oxidative stress, endothelial dysfunction, and the acceleration of atherogenesis [30], all of which may cause the progression of atherosclerosis and, consequently, nephrosclerosis. On the basis of this mechanism, resting heart rate can be regarded as a predictor of both cardiovascular and renal outcomes [31–33]. The interactions among heart rate, kidney dysfunction, and risk of cardiovascular disease can create a vicious cycle that increases the risk of cardiovascular complications in patients with pre-existing cardiovascular disease.

In a health evaluation program, subjects with a high heart rate experienced a greater decrease in their eGFR and exhibited a higher odds ratio of developing proteinuria [31]. The prevalence of microalbuminuria is linked to heart rate in patients with hypertension who exhibit cardiovascular risk factors [34]. Patients with type 2 diabetes mellitus who have a higher resting heart rate also exhibit a higher incidence of new-onset or progressive nephropathy and retinopathy [35]. The present study demonstrates a correlation between increased heart rate and accelerated kidney function deterioration, as well as a higher likelihood of adverse kidney events following ischemic stroke.

However, the mechanisms affecting heart rate may differ between atrial fibrillation and normal sinus rhythm. In the RACE II trial, the degree of heart rate control did not significantly impact major cardiovascular events in patients with atrial fibrillation [36]. Atrial fibrillation was present in 13% of subjects in this study, with a higher prevalence in those with elevated heart rates, particularly in the ≥90 bpm group. This suggests that atrial fibrillation may have contributed to the higher heart rates observed and highlights the need to consider differences between sinus rhythm and atrial fibrillation when interpreting the association between heart rate and renal outcomes. Our subgroup analysis showed that the link between heart rate and composite renal outcomes was significant only in patients without atrial fibrillation (Fig. 5), and the association with eGFR decline was weaker in those with atrial fibrillation (Supplementary Table S4 and Supplementary Fig. S2). This suggests that heart rate may have a greater impact on renal outcomes in patients without atrial fibrillation, warranting further study in those with atrial fibrillation.

In the present study, the rate of eGFR decline was faster with a higher baseline eGFR and slower with a lower baseline eGFR (Fig. 3). This finding is consistent with that of another study; i.e. the rate of eGFR decline depended mainly on baseline eGFR [37]. Although the reason for the slower rate of eGFR decline in patients with lower baseline eGFR is unknown, declining kidney function might activate a compensatory mechanism. In this study, the patients across the heart rate subgroups did not differ significantly in terms of their risk of composite renal outcomes when they had an eGFR of <60 mL/min/1.73 m^2^ (Table 3), suggesting that the role of a patient's heart rate in the deterioration of their renal function is less relevant at this stage.

Although the subgroup with a lower heart rate seems to have a lower baseline eGFR value, the baseline eGFR is influenced by many other factors, such as age, sex, and various cardiovascular risk factors, in addition to heart rate. The baseline eGFR reflects the result of interactions among these factors. Therefore, without considering other confounding factors, it is not possible to accurately determine the specific impact of heart rate on baseline eGFR.

An individual's heart rate is controlled by the activity of their autonomic nervous system, which comprises the sympathetic and parasympathetic nervous systems. An elevated heart rate may result from increased sympathetic activity or reduced parasympathetic activity. The role of the renal sympathetic nerves in controlling renal function has been extensively explored. The sympathetic system innervates all segments of the kidneys, including the blood vessels and tubules [38]. Increased renal sympathetic nerve activity causes increased renin secretion, increased renal tubular sodium reabsorption, and decreased renal blood flow [39]. Elevated sympathetic nerve activity not only exacerbates hypertension but also plays a detrimental role in the progression of CKD independent of increased blood pressure [40]. Although β-blockers provide several positive effects that hinder overactivity of the sympathetic nerve, the role of β-blockers in renal protection remains a controversial topic. The reduced cardiac output and consequent alteration of kidney perfusion caused by β-blockers are regarded as harmful for patients with CKD [41].

Ivabradine is a heart rate-lowering agent that selectively and specifically inhibits cardiac pacemaker current (*I*_f_). Although in patients with chronic stable systolic heart failure heart rate is directly and independently associated with the risk of worsening renal function, the reduction in heart rate due to ivabradine use was reported to have a neutral effect on renal function in a study with 2 years of follow-up [42]. The complex and bidirectional interactions between the renin–angiotensin system and autonomic nervous system have been extensively examined. Although ACEIs and ARBs have been demonstrated to delay the progression of CKD [43], ACEI or ARB use in very early nephropathy (i.e. an eGFR of >60 mL/min with microalbuminuria or normoalbuminuria regardless of diabetes status) has not been demonstrated to provide clear advantages [44], and most patients with stroke belong to this population.

In addition to sympathetic innervation, the kidneys also receive parasympathetic innervation through the small renal branches of the vagus nerve. Although the role of parasympathetic fibers in the kidneys remains unclear, Inoue *et al*. employed an ischemia/reperfusion model of acute kidney injury to demonstrate the positive effect of vagal stimulation [45]. The chronic administration of cholinesterase inhibitors has been demonstrated to induce parasympathetic activation [46]. In patients with Alzheimer disease who undergo routine care, the use of cholinesterase inhibitors (versus non-use) was reported to be associated with a lower risk of CKD progression [47].

The present study has several limitations that should be acknowledged. First, as our cohort consists exclusively of Asian patients with ischemic stroke from a single healthcare system in Taiwan, further research across diverse ethnicities, disease entities, and healthcare systems is warranted to determine the broader applicability of these findings and to assess potential variations in outcomes among different populations. Second, most of the patients included in the present study were older adults (interquartile age range, 59–76 years), which limited the generalizability of our results to other age groups. Third, the availability of plasma creatinine measurements used to construct eGFR slopes varied among the included patients. Fourth, vital signs, including heart rate, were averaged during the acute stroke period. The acute-phase data may be influenced by sympathetic activation, infection, or treatments, and may not reflect the chronic phase. Future studies should aim to include data from both acute and chronic phases to provide a more comprehensive understanding of the long-term impact of heart rate on eGFR decline in ischemic stroke patients. Finally, although our observational design identified associations between resting heart rate and renal function decline, it cannot confirm causality. Despite efforts to minimize biases, a prospective study would provide stronger evidence for causal relationships, and future research should aim to validate these findings.

In conclusion, initial in-hospital heart rate was revealed to be independently associated with the rate of eGFR decline and risk of composite renal outcomes, particularly in patients without atrial fibrillation. Although heart rate is the least invasive and most easily accessible biomarker, it is usually not a component of the risk assessment conducted for patients who experienced ischemic stroke. Clinicians managing ischemic stroke patients should consider heart rate monitoring as part of their routine assessment to potentially mitigate eGFR decline. Regular heart rate measurement, particularly during the acute phase of stroke in patients without atrial fibrillation, may help identify patients at risk for rapid eGFR decline. Further studies are needed to explore this relationship specifically in patients with atrial fibrillation and in diverse populations. Incorporating targeted interventions, such as cholinesterase inhibitors, could potentially mitigate the adverse effects of elevated heart rate on renal function. Such research could ultimately lead to enhanced multidisciplinary care strategies that encompass cardiovascular and renal protection in stroke patients.

## Supplementary Material

sfae387_Supplemental_File

## Data Availability

The data that support the findings of this study are available from Chang Gung Research Databank at Chang Gung Memorial Hospital, Chiayi Branch, but restrictions apply to the availability of these data, which were used under license for the current study, and so are not publicly available. Data are, however, available from the authors upon reasonable request and with permission of the local Institutional Review Board of Chang Gung Memorial Hospital, Chiayi Branch, Taiwan.

## References

[1] Wu CL, Tsai CC, Kor CT et al. Stroke and risks of development and progression of kidney diseases and end-stage renal disease: a nationwide population-based cohort study. PLoS One 2016;11:e0158533. 10.1371/journal.pone.0158533 27355475 PMC4927175

[2] Ghoshal S, Freedman BI. Mechanisms of stroke in patients with chronic kidney disease. Am J Nephrol 2019;50:229–39. 10.1159/000502446 31461699

[3] Hayden D, McCarthy C, Akijian L et al. Renal dysfunction and chronic kidney disease in ischemic stroke and transient ischemic attack: a population-based study. Int J Stroke 2017;12:761–9. 10.1177/1747493017701148 28643553

[4] Gansevoort RT, Correa-Rotter R, Hemmelgarn BR et al. Chronic kidney disease and cardiovascular risk: epidemiology, mechanisms, and prevention. The Lancet 2013;382:339–52. 10.1016/S0140-6736(13)60595-4 23727170

[5] Masson P, Webster AC, Hong M et al. Chronic kidney disease and the risk of stroke: a systematic review and meta-analysis. Nephrol Dial Transplant 2015;30:1162–9. 10.1093/ndt/gfv009 25681099

[6] Ito S, Nagasawa T, Abe M et al. Strain vessel hypothesis: a viewpoint for linkage of albuminuria and cerebro-cardiovascular risk. Hypertens Res 2009;32:115–21. 10.1038/hr.2008.27 19262469

[7] Giannoglou GD, Chatzizisis YS, Zamboulis C et al. Elevated heart rate and atherosclerosis: an overview of the pathogenetic mechanisms. Int J Cardiol 2008;126:302–12. 10.1016/j.ijcard.2007.08.077 18068835

[8] Seravalle G, Quarti-Trevano F, Vanoli J et al. Autonomic cardiovascular alterations as therapeutic targets in chronic kidney disease. Clin Auton Res 2021;31:491–8. 10.1007/s10286-021-00786-6 33606138 PMC8292281

[9] Whelton SP, Narla V, Blaha MJ et al. Association between resting heart rate and inflammatory biomarkers (high-sensitivity C-reactive protein, interleukin-6, and fibrinogen) (from the Multi-Ethnic Study of Atherosclerosis). Am J Cardiol 2014;113:644–9. 10.1016/j.amjcard.2013.11.009 24393259 PMC4280910

[10] Zoccali C, Leonardis D, Enia G et al. Heart rate, age and the risk of progression to kidney failure in patients with CKD. J Nephrol 2012;25(Suppl 19):20–7. 10.5301/jn.5000229 23032914

[11] Tsai MK, Gao W, Chien KL et al. Resting heart rate independent of cardiovascular disease risk factors is associated with end-stage renal disease: a cohort study based on 476 347 adults. J Am Heart Assoc 2023;12:e030559. 10.1161/JAHA.123.030559 38038184 PMC10727324

[12] Strittmatter M, Meyer S, Fischer C et al. Location-dependent patterns in cardio-autonomic dysfunction in ischaemic stroke. Eur Neurol 2003;50:30–8. 10.1159/000070856 12824710

[13] Johns EJ, Kopp UC, DiBona GF. Neural control of renal function. Compr Physiol 2011;1:731–67. 10.1002/cphy.c100043 23737201

[14] Zhao M, Guan L, Wang Y. The association of autonomic nervous system function with ischemic stroke, and treatment strategies. Front Neurol 2019;10:1411. 10.3389/fneur.2019.01411 32038467 PMC6987371

[15] Custodis F, Baumhakel M, Schlimmer N et al. Heart rate reduction by ivabradine reduces oxidative stress, improves endothelial function, and prevents atherosclerosis in apolipoprotein E-deficient mice. Circulation 2008;117:2377–87. 10.1161/CIRCULATIONAHA.107.746537 18443241

[16] Tsai MS, Lin MH, Lee CP et al. Chang Gung Research Database: a multi-institutional database consisting of original medical records. Biomed J 2017;40:263–69. 10.1016/j.bj.2017.08.002 29179881 PMC6138604

[17] Lee JD, Kuo YW, Lee CP et al. Initial in-hospital heart rate is associated with long-term survival in patients with acute ischemic stroke. Clin Res Cardiol 2022;111:651–62. 10.1007/s00392-021-01953-5 34687320 PMC9151537

[18] Sung SF, Hsieh CY, Lin HJ et al. Validity of a stroke severity index for administrative claims data research: a retrospective cohort study. BMC Health Serv Res 2016;16:509. 10.1186/s12913-016-1769-8 27660046 PMC5034530

[19] Levey AS, Stevens LA, Schmid CH et al. A new equation to estimate glomerular filtration rate. Ann Intern Med 2009;150:604–12. 10.7326/0003-4819-150-9-200905050-00006 19414839 PMC2763564

[20] Pickering TG, Hall JE, Appel LJ et al. Recommendations for blood pressure measurement in humans and experimental animals: part 1: blood pressure measurement in humans: a statement for professionals from the Subcommittee of Professional and Public Education of the American Heart Association Council on High Blood Pressure Research. Circulation 2005;111:697–716. 15699287 10.1161/01.CIR.0000154900.76284.F6

[21] Li HY, Wu YL, Tu ST et al. Trends of mortality in diabetic patients in Taiwan: a nationwide survey in 2005-2014. J Formosan Med Assoc 2019;118(Suppl 2):S83–9. 10.1016/j.jfma.2019.07.008 31351690

[22] Shou H, Hsu JY, Xie D et al. Analytic considerations for repeated measures of eGFR in cohort studies of CKD. Clin J Am Soc Nephrol 2017;12:1357–65. 10.2215/CJN.11311116 28751576 PMC5544518

[23] Jonsson AJ, Lund SH, Eriksen BO et al. Incidence of and risk factors of chronic kidney disease: results of a nationwide study in Iceland. Clin Kidney J 2022;15:1290–99. 10.1093/ckj/sfac051 35756731 PMC9217641

[24] Zhao Q, Yan T, Chopp M et al. Brain-kidney interaction: renal dysfunction following ischemic stroke J Cereb Blood Flow Metab 2020;40:246–62. 10.1177/0271678X19890931 31766979 PMC7370616

[25] Agarwal A, Cheung AK, Ma J et al. Effect of baseline kidney function on the risk of recurrent stroke and on effects of intensive blood pressure control in patients with previous lacunar stroke: a post hoc analysis of the SPS3 Trial (Secondary Prevention of Small Subcortical Strokes). J Am Heart Assoc 2019;8:e013098. 10.1161/JAHA.119.013098 31423869 PMC6759889

[26] Wang IK, Lien LM, Lee JT et al. Renal dysfunction increases the risk of recurrent stroke in patients with acute ischemic stroke. Atherosclerosis 2018;277:15–20. 10.1016/j.atherosclerosis.2018.07.033 30170219

[27] Chang HC, Huang CJ, Yang AC et al. Role of heart rate variability in association between glomerular hyperfiltration and all-cause mortality. J Am Heart Assoc 2021;10:e021585. 10.1161/JAHA.121.021585 34889105 PMC9075221

[28] Delacroix S, Chokka RG, Nelson AJ et al. Renal sympathetic denervation increases renal blood volume per cardiac cycle: a serial magnetic resonance imaging study in resistant hypertension. Int J Nephrol Renovasc Dis 2017;10:243–49. 10.2147/IJNRD.S131220 28919800 PMC5587163

[29] Navar LG . Physiology: hemodynamics, endothelial function, renin-angiotensin-aldosterone system, sympathetic nervous system. J Am Soc Hypertens 2014;8:519–24. 10.1016/j.jash.2014.05.014 25064774 PMC4115246

[30] Custodis F, Schirmer SH, Baumhakel M et al. Vascular pathophysiology in response to increased heart rate. J Am Coll Cardiol 2010;56:1973–83. 10.1016/j.jacc.2010.09.014 21126638

[31] Inoue T, Iseki K, Iseki C et al. Heart rate as a risk factor for developing chronic kidney disease: longitudinal analysis of a screened cohort. Clin Exp Nephrol 2009;13:487–93. 10.1007/s10157-009-0193-3 19444548

[32] Bohm M, Schumacher H, Schmieder RE et al. Resting heart rate is associated with renal disease outcomes in patients with vascular disease: results of the ONTARGET and TRANSCEND studies. J Intern Med 2015;278:38–49. 10.1111/joim.12333 25431275

[33] Lonn EM, Rambihar S, Gao P et al. Heart rate is associated with increased risk of major cardiovascular events, cardiovascular and all-cause death in patients with stable chronic cardiovascular disease: an analysis of ONTARGET/TRANSCEND. Clin Res Cardiol 2014;103:149–59. 10.1007/s00392-013-0644-4 24356937

[34] Bohm M, Thoenes M, Neuberger HR et al. Atrial fibrillation and heart rate independently correlate to microalbuminuria in hypertensive patients. Eur Heart J 2009;30:1364–71. 10.1093/eurheartj/ehp124 19383737

[35] Hillis GS, Hata J, Woodward M et al. Resting heart rate and the risk of microvascular complications in patients with type 2 diabetes mellitus. J Am Heart Assoc 2012;1:e002832. 10.1161/JAHA.112.002832 23316296 PMC3541618

[36] Van Gelder IC, Groenveld HF, Crijns HJ et al. Lenient versus strict rate control in patients with atrial fibrillation. N Engl J Med 2010;362:1363–73. 10.1056/NEJMoa1001337 20231232

[37] Baba M, Shimbo T, Horio M et al. Longitudinal study of the decline in renal function in healthy subjects. PLoS One 2015;10:e0129036. 10.1371/journal.pone.0129036 26061083 PMC4464887

[38] DiBona GF . Neural control of the kidney: functionally specific renal sympathetic nerve fibers. Am J Physiol Regul Integr Comp Physiol 2000;279:R1517–24. 10.1152/ajpregu.2000.279.5.R1517 11049831

[39] Wyss JM, Carlson SH. The role of the central nervous system in hypertension. Curr Hypertens Res 1999;1:246–53. 10.1007/s11906-999-0029-2 10981074

[40] Grassi G, Quarti-Trevano F, Seravalle G et al. Early sympathetic activation in the initial clinical stages of chronic renal failure. Hypertension 2011;57:846–51. 10.1161/HYPERTENSIONAHA.110.164780 21300663

[41] Ptinopoulou AG, Pikilidou MI, Lasaridis AN. The effect of antihypertensive drugs on chronic kidney disease: a comprehensive review. Hypertens Res 2013;36:91–101. 10.1038/hr.2012.157 23051659

[42] Voors AA, van Veldhuisen DJ, Robertson M et al. The effect of heart rate reduction with ivabradine on renal function in patients with chronic heart failure: an analysis from SHIFT. Eur J Heart Fail 2014;16:426–34. 10.1002/ejhf.59 24504937

[43] Xie X, Liu Y, Perkovic V et al. Renin-angiotensin system inhibitors and kidney and cardiovascular outcomes in patients with CKD: a Bayesian network meta-analysis of randomized clinical trials. Am J Kidney Dis 2016;67:728–41. 10.1053/j.ajkd.2015.10.011 26597926

[44] Bakris GL . ACE inhibitors and ARBs: are they better than other agents to slow nephropathy progression? J Clin Hypertens 2007;9:413–5. 10.1111/j.1524-6175.2007.07234.x PMC8110074 17541325

[45] Inoue T, Rosin DL, Okusa MD. CAPing inflammation and acute kidney injury. Kidney Int 2016;90:462–5. 10.1016/j.kint.2016.07.009 27521104

[46] Umegaki H, Khookhor O. The response of the autonomic nervous system to the cholinesterase inhibitor, donepezil. Neuro Endocrinol Lett 2013;34:383–7. 23922048

[47] Xu H, Garcia-Ptacek S, Bruchfeld A et al. Association between cholinesterase inhibitors and kidney function decline in patients with Alzheimer's dementia. Kidney Int 2023;103:166–76. 10.1016/j.kint.2022.09.022 36341731

