# Supplementary material for: Impact of heart rate on eGFR decline in ischemic stroke patients
Source: Clin Kidney J. 2024 Nov 30;18(1):sfae387. doi: 10.1093/ckj/sfae387 (PMC11744309; doi:10.1093/ckj/sfae387)
Supplement: sfae387_Supplemental_File [file sfae387_supplemental_file.pdf]

Supplementary Table S1. Association of heart rate with annualized change in eGFR

	Annualized Change in eGFR (95% Confidence Interval)							
**Heart Rate**	**Unadjusted**	*P* value	**Model 1**	*P* value	**Model 2**	*P* value	**Model 3**	*P* value
<60 bpm	−2.149 (−2.488 to −1.810)	<0.001	−2.182 (−2.519 to −1.846)	<0.001	−2.136 (−2.469 to −1.804)	<0.001	−2.123 (−2.456 to −1.790)	<0.001
60–69 bpm	−2.457 (−2.660 to −2.254)	<0.001	−2.531 (−2.730 to −2.332)	<0.001	−2.496 (−2.691 to −2.300)	<0.001	−2.487 (−2.682 to −2.291)	<0.001
70–79 bpm	−2.772 (−2.949 to −2.594)	<0.001	−2.856 (−3.034 to −2.678)	<0.001	−2.846 (−3.021 to −2.670)	<0.001	−2.831 (−3.007 to −2.656)	<0.001
80–89 bpm	−3.257 (−3.612 to −2.902)	<0.001	−3.455 (−3.812 to −3.098)	<0.001	−3.364 (−3.712 to −3.016)	<0.001	−3.345 (−3.693 to −2.998)	<0.001
≥90 bpm	−3.905 (−4.599 to −3.212)	<0.001	−3.969 (−4.650 to −3.288)	<0.001	−3.909 (−4.577 to −3.241)	<0.001	−3.898 (−4.568 to −3.227)	<0.001

Model 1 is adjusted for age and sex.

Model 2 is adjusted for the variables in model 1 plus stroke severity, body mass index, hypertension, diabetes, dyslipidemia, atrial fibrillation, coronary artery disease, congestive heart failure, history of cancer, smoking status, total cholesterol, triglycerides, alanine aminotransferase, glycated hemoglobin, systolic blood pressure, and diastolic blood pressure.

Model 3 is adjusted for the variables in model 2 plus angiotensin converting enzyme Inhibitor / angiotensin receptor blocker use, antithrombotic use, beta-blocker use, parasympathomimetic use, and statin use.

Abbreviations: eGFR, estimated glomerular filtration rate; bpm, beats per minute.

Supplementary Table S2. Association of heart rate with annualized change of eGFR according to baseline eGFR strata

	Annualized Change in eGFR (95% Confidence Interval)					
**Heart Rate**	**eGFR ≥90**	*P* value	**60≤ eGFR <90**	*P* value	**30≤ eGFR <60**	*P* value
<60 bpm	−2.731 (−3.174 to −2.288)	<0.001	−2.022 (−2.487 to −1.556)	<0.001	−1.204 (−2.060 to −0.349)	0.006
60–69 bpm	−2.770 (−3.057 to −2.483)	<0.001	−2.385 (−2.656 to −2.114)	<0.001	−1.875 (−2.379 to −1.370)	<0.001
70–79 bpm	−3.198 (−3.466 to −2.929)	<0.001	−2.695 (−2.969 to −2.421)	<0.001	−1.896 (−2.275 to −1.516)	<0.001
80–89 bpm	−3.782 (−4.286 to −3.277)	<0.001	−2.929 (−3.404 to −2.455)	<0.001	−2.343 (−3.167 to −1.519)	<0.001
≥90 bpm	−4.531 (−5.520 to −3.543)	<0.001	−2.989 (−3.949 to −2.029)	<0.001	−2.167 (−3.451 to −0.883)	0.001

Effects of mean heart rate level, years and their interaction are adjusted for age, sex, stroke severity, body mass index, hypertension, diabetes, dyslipidemia, atrial fibrillation, coronary artery disease, congestive heart failure, history of cancer, smoking status, total cholesterol, triglycerides, alanine aminotransferase, glycated hemoglobin, systolic blood pressure, diastolic blood pressure, angiotensin converting enzyme Inhibitor / angiotensin receptor blocker use, antithrombotic use, beta-blocker use, parasympathomimetic use and statin use.

Abbreviations: eGFR, estimated glomerular filtration rate; bpm, beats per minute.

Supplementary Table S3. Hazard ratios of model variables for developing composite renal outcomes

Variables	Hazard ratio	95% CI	*P* value
Mean heart rate level (ref: <60 bpm)			
≥60 and <70 bpm	1.17	0.89–1.53	0.3
≥70 and <80 bpm	1.54	1.19–2.00	0.001
≥80 and <90 bpm	1.72	1.30–2.27	<0.001
≥90 bpm	1.84	1.31–2.57	<0.001
Age, year	1.01	1.01–1.02	<0.001
Male (ref: female)	1.20	1.04–1.39	0.01
eNIHSS	1.02	1.00–1.03	0.04
Body mass index (ref: ≥18.5, <24)			
<18.5	1.07	0.64–1.78	0.8
≥24, <27	0.88	0.73–1.07	0.2
≥27, <30	0.82	0.65–1.03	0.09
≥30	0.74	0.56–0.98	0.04
missing	0.92	0.78–1.08	0.3
Hypertension (ref: without hypertension)	1.02	0.86–1.22	0.8
DM (ref: without DM)	1.95	1.67–2.28	<0.001
Dyslipidemia (ref: without dyslipidemia)	0.92	0.80–1.06	0.2
AF (ref: without AF)	1.09	0.89–1.33	0.4
CAD (ref: without CAD)	1.37	1.13–1.66	0.001
CHF (ref: without CHF)	1.36	1.03–1.80	0.03
Cancer (ref: without Cancer)	1.64	1.30–2.08	<0.001
Smoker (ref: non-smoker)	1.25	1.08–1.45	0.003
Total cholesterol (ref: Q1)			
Q2	0.87	0.73–1.04	0.1
Q3	0.90	0.75–1.07	0.2
Q4	1.16	0.96–1.40	0.1
missing	0.00	0.00–Inf	0.9
Triglyceride (ref: Q1)			
Q2	0.98	0.81–1.18	0.8
Q3	1.14	0.94–1.39	0.2
Q4	1.53	1.26–1.85	<0.001
missing	NA	NA–NA	NA
ALT (ref: Q1)			
Q2	0.84	0.71–0.99	0.03
Q3	0.72	0.61–0.85	<0.001
Q4	0.69	0.58–0.82	<0.001
missing	0.46	0.15–1.43	0.2
HbA1c (ref: Q1)			
Q2	1.05	0.80–1.39	0.7
Q3	0.92	0.69–1.22	0.6

Q4	1.56	1.19–2.05	0.001
missing	1.29	1.03–1.62	0.03
Mean SBP (ref: <130 mmHg)			
≥130, <140 mmHg	1.30	0.97–1.75	0.08
≥140, <150 mmHg	1.48	1.10–1.98	0.01
≥150, <160 mmHg	1.90	1.40–2.58	<0.001
≥160 mmHg	2.91	2.13–3.98	<0.001
missing	0.34	0.02–5.57	0.4
Mean DBP (ref: <70 mmHg)			
≥70, <80 mmHg	1.01	0.72–1.40	0.9
≥80, <90 mmHg	0.75	0.54–1.06	0.1
≥90, <100 mmHg	0.70	0.49–1.02	0.06
≥100 mmHg	0.58	0.38–0.89	0.01
missing	2.21	0.30–16.25	0.4
ACEI/ARB use (ref: no use)	1.24	1.07–1.44	0.004
Antithrombotic use (ref: no use)	0.87	0.60–1.24	0.4
Beta blocker use (ref: no use)	1.19	1.05–1.36	0.007
Parasympathomimetic use (ref: no use)	1.05	0.77–1.43	0.8
Statin use (ref: no use)	1.00	0.87–1.15	0.9

Abbreviations: CI, confidence interval; ref, reference; eNIHSS, estimated National Institutes of Health Stroke Scale; DM, diabetes mellitus; AF, atrial fibrillation; CAD, coronary artery disease; CHF, congestive heart failure; Q, quartile; Inf, infinity; NA, not available; ALT, alanine aminotransferase; HbA1c, glycated hemoglobin; SBP, systolic blood pressure; DBP, diastolic blood pressure; ACEI, angiotensin converting enzyme Inhibitor; ARB, angiotensin receptor blocker.

Supplementary Table S4. Annualized change of average eGFR across heart rate subgroup in patients with and without atrial fibrillation

**With atrial fibrillation (N = 1231)**	Unadjusted model	*P*	Model 1	*P*	Model 2	*P*	Model 3	*P*
**Heart rate <60 bpm**	-2.064 (-3.270 to -0.857)	0.001	-2.125 (-3.525 to -0.725)	0.003	-1.988 (-3.215 to -0.762)	0.002	-1.915 (-3.102 to -0.728)	0.002
**Heart rate ≥60 and <70 bpm**	-2.197 (-2.787 to -1.606)	<0.001	-2.269 (-2.847 to -1.690)	<0.001	-2.154 (-2.724 to -1.584)	<0.001	-2.107 (-2.675 to -1.540)	<0.001
**Heart rate ≥70 and <80 bpm**	-1.958 (-2.416 to -1.500)	<0.001	-2.116 (-2.575 to -1.658)	<0.001	-2.102 (-2.551 to -1.653)	<0.001	-2.088 (-2.536 to -1.641)	<0.001
**Heart rate ≥80 and <90 bpm**	-2.459 (-3.188 to -1.730)	<0.001	-2.669 (-3.363 to -1.975)	<0.001	-2.594 (-3.273 to -1.914)	<0.001	-2.593 (-3.268 to -1.918)	<0.001
**Heart rate ≥90 bpm**	-2.501 (-3.703 to -1.299)	<0.001	-2.634 (-3.835 to -1.433)	<0.001	-2.601 (-3.801 to -1.402)	<0.001	-2.599 (-3.752 to -1.447)	<0.001
**Without atrial fibrillation (N = 8135)**	Unadjusted model	*P*	Model 1	*P*	Model 2	*P*	Model 3	*P*
**Heart rate <60 bpm**	-2.154 (-2.511 to -1.797)	<0.001	-2.185 (-2.536 to -1.833)	<0.001	-2.143 (-2.490 to -1.797)	<0.001	-2.135 (-2.481 to -1.788)	<0.001
**Heart rate ≥60 and <70 bpm**	-2.478 (-2.693 to -2.263)	<0.001	-2.547 (-2.758 to -2.337)	<0.001	-2.512 (-2.718 to -2.306)	<0.001	-2.505 (-2.711 to -2.299)	<0.001
**Heart rate ≥70 and <80 bpm**	-2.861 (-3.052 to -2.671)	<0.001	-2.931 (-3.121 to -2.740)	<0.001	-2.912 (-3.099 to -2.725)	<0.001	-2.898 (-3.085 to -2.711)	<0.001
**Heart rate ≥80 and <90 bpm**	-3.390 (-3.790 to -2.991)	<0.001	-3.574 (-3.976 to -3.172)	<0.001	-3.482 (-3.873 to -3.092)	<0.001	-3.462 (-3.852 to -3.072)	<0.001
**Heart rate ≥90 bpm**	-4.391 (-5.255 to -3.527)	<0.001	-4.430 (-5.267 to -3.594)	<0.001	-4.364 (-5.176 to -3.553)	<0.001	-4.363 (-5.178 to -3.549)	<0.001

Model 1 is adjusted for age and sex.

Model 2 is adjusted for the variables in model 1 plus stroke severity, body mass index, hypertension, diabetes, dyslipidemia, atrial fibrillation, coronary artery disease, congestive heart failure, history of cancer, smoking status, total cholesterol, triglycerides, ALT, HbA1c, mean SBP, and mean DBP.

Model 3 is adjusted for the variables in model 2 plus ACEI/ARB use, antithrombotics use, beta-blocker use, parasympathomimetics use and statin use.

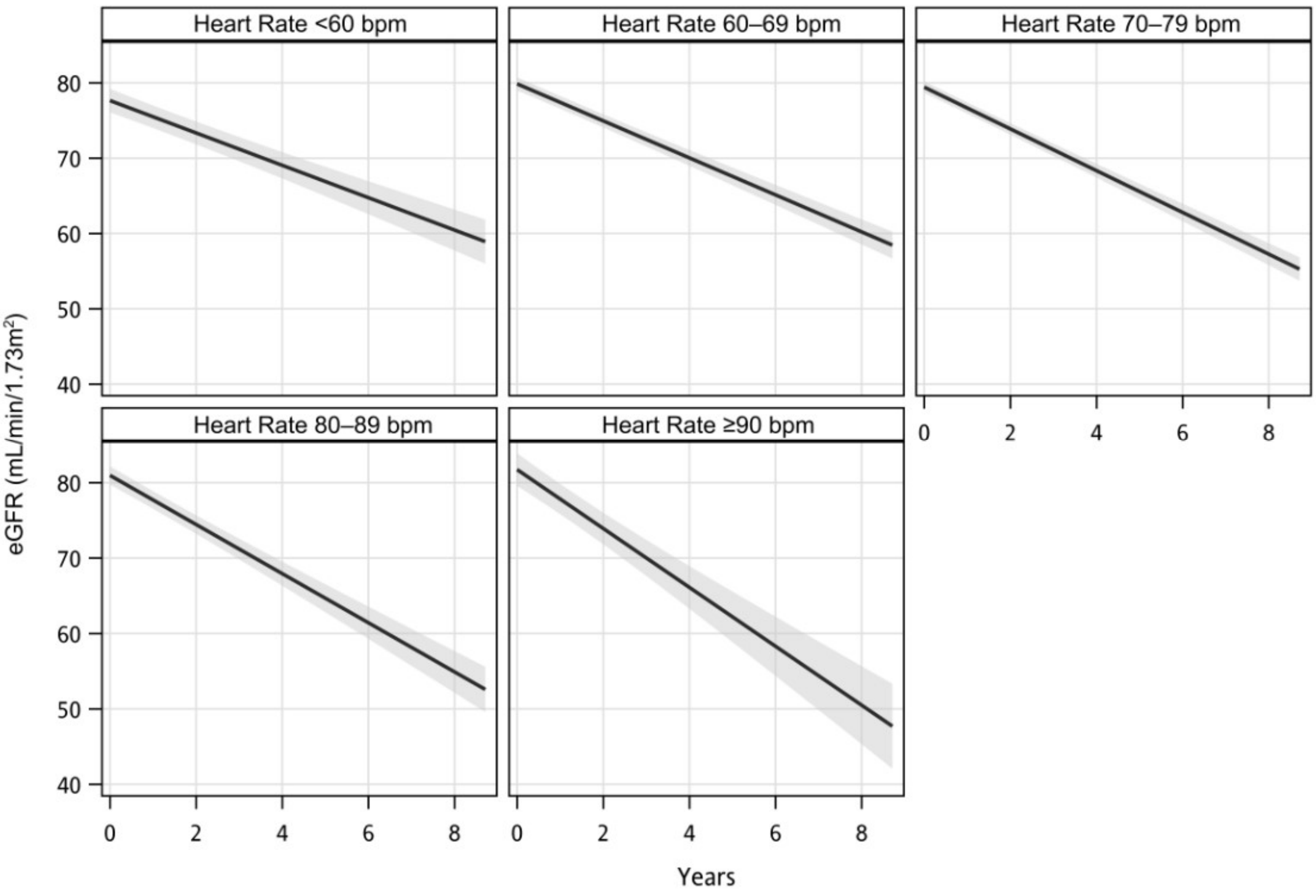

Supplementary Figure S1. Longitudinal evolution of eGFR in different heart rate subgroups.

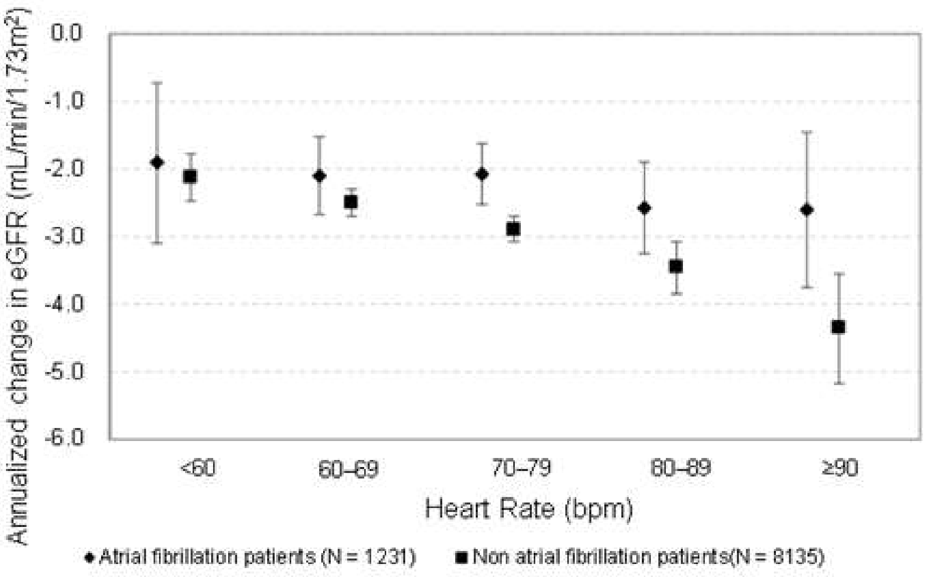

Supplementary Figure S2. Annualized change of average eGFR across heart rate subgroup in patients with and without atrial fibrillation
